# Variably protease‐sensitive prionopathy mimicking frontotemporal dementia

**DOI:** 10.1111/neup.12538

**Published:** 2019-03-07

**Authors:** Miren Aizpurua, Sashika Selvackadunco, Helen Yull, Christopher M. Kipps, James W. Ironside, Istvan Bodi

**Affiliations:** ^1^ Clinical Neuropathology King's College Hospital, NHS Foundation Trust London UK; ^2^ London Neurodegenerative Diseases Brain Bank IOPPN London UK; ^3^ National Creutzfeldt‐Jakob Disease Research & Surveillance Unit, Centre for Clinical Brain Sciences University of Edinburgh Edinburgh UK; ^4^ Wessex Neurological Centre University Hospital Southampton NHS Foundation Trust Southampton UK; ^5^ Clinical Neurosciences University of Southampton Southampton UK

**Keywords:** Creutzfeldt–Jakob disease, prions, prion diseases, prion protein, variably protease‐sensitive prionopathy

## Abstract

Sporadic prion diseases are fatal neurodegenerative disorders characterized clinically by rapidly progressive dementia and myoclonus. Variably protease‐sensitive prionopathy (VPSPr) is a recently identified sporadic human prion disorder that may present with a lengthy atypical clinical history. Here, we describe a case of VPSPr in a patient with a long history of suspected frontotemporal dementia (FTD). A 61‐year‐old man presented with speech difficulties, including naming objects and constructing multipart sentences, while there was no difficulty in comprehension. Movement abnormalities included slightly jerky pursuit, minor dysmetria of saccades and brisk reflexes. There was no family history of dementia. Later he developed swallowing difficulties and the possibility of FTD with motor neuron disease was suspected. He died at the age of 71 and his brain was donated to the London Neurodegenerative Diseases Brain Bank. The brain (1004 g) showed mild to moderate atrophy, predominantly in the frontal lobe. Histology revealed moderate spongiform microvacuolation mostly affecting the frontal and parietal cortices, but also present focally in the basal ganglia and the cerebellum. Only mild Alzheimer pathology was found by extensive immunohistochemistry, in keeping with BrainNet Europe stage II. Trans‐activation response DNA‐binding protein 43 kDa and α‐synuclein immunostains were negative. Immunostaining for prion protein (PrP) showed granular/synaptic positivity in a patchy distribution, mainly within the deeper cortex, and also revealed microplaques in the cerebellum and basal ganglia. Western blotting confirmed a low molecular weight protease‐resistant PrP band with a faint ladder‐like pattern in the absence of types 1 and 2 isoforms. These features are diagnostic of VPSPr. VPSPr can mimic various neurodegenerative conditions; diagnosis requires both PrP immunohistochemistry and Western blotting. The presence of patchy spongiform change in the absence of other neurodegenerative pathology should raise suspicion of VPSPr, even in elderly patients with a lengthy clinical history.

## INTRODUCTION

Human prion diseases are fatal neurodegenerative disorders that are characterized neuropathologically by the abnormal accumulation of a misfolded prion protein (PrP) in the central nervous system. The mechanism by which the cellular PrP (PrP^C^) is converted into the pathogenic scrapie‐type PrP (PrP^Sc^) appears to involve a post‐translational change in PrP^C^ conformation, from a predominantly α‐helical into a predominantly β‐sheet structure.^1^ PrP^Sc^ is the major, if not the sole, component of the transmissible agent in prion diseases. PrP^Sc^ is relatively insoluble and aggregates extracellularly, possibly inducing adjacent tissue malfunction, although the exact pathological mechanism is still unclear. Human prion diseases differ not only in their clinical and neuropathological features, but also in the biochemical features (differential glycosylation and relatively resistance to proteinase digestion) of PrP^Sc^ in the brain, making prionopathies a much more heterogeneous group of disorders than initially considered.

Human prion diseases are classified into three main categories: sporadic, acquired or genetic (familial). Sporadic Creutzfeldt–Jakob disease (sCJD) is the most common human prion disease, occurring world‐wide and characterized clinically by rapidly progressive dementia and myoclonus. However, sCJD is known to be clinically and pathologically heterogeneous, and is currently sub‐classified into six subtypes.^2^ The molecular basis for this heterogeneity is thought to correspond to an interaction between the naturally occurring methionine/valine polymorphism at codon 129 of the prion protein gene (*PRNP*) (MM, MV, or VV) and the abnormal prion protein isoform in the brain, as determined by Western blot analysis of the protease‐resistant band (termed PrP^res^) obtained by treating PrP^Sc^ with protease K. The resulting bands are then classified by their molecular weight as type 1 or type 2.^3^, ^4^ Recent investigations have identified another sporadic human prion disease, termed variably protease‐sensitive prionopathy (VPSPr) which lacks *PRNP* mutations but is clinically and biochemically different from sporadic CJD.^5^ Here, we report a case of VPSPr in a UK patient with a lengthy clinical history mimicking frontotemporal dementia.

## CLINICAL SUMMARY

**Figure 1:**
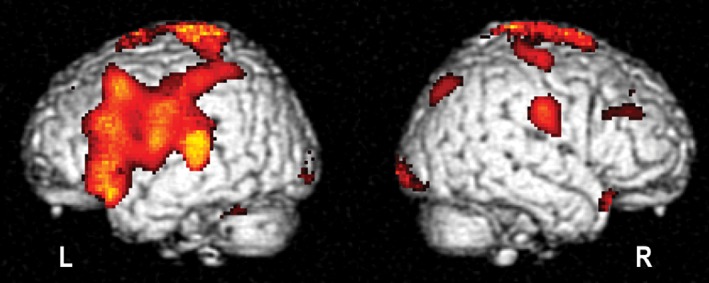
99mTc – hexylmethylpropylene amineoxine – single‐photon emission computed tomography scan of subject's cerebral perfusion compared against age‐matched controls, rendered on template brain at *P* < 0.01 threshold. Scan shows predominant left dorsolateral prefrontal cortex and insula hypoperfusion consistent with a non‐fluent aphasic syndrome.

Brain donation to the London Neurodegenerative Diseases Brain Bank for research was received from a 71‐year‐old man. He presented at the age of 69 with 8 years history of speech difficulties. He apparently had a stressful time some years ago when selling his company and noticed that his speech was less fluent and he became prone to making dysphasic errors. He also noticed a slight change in handwriting. Although the onset of the disease was very slow, his partner did highlight a substantial change in his attention, some degree of repetitive questioning and memory disturbances such as loosing or misplacing objects and forgetting the names of people and objects. He lost track of his thoughts if he was interrupted; however, there was no topographical disorientation. He had no difficulty with self‐care and there were no mood or behavioral disturbance, although a slight reduction in motivation was observed. No visual hallucinations were reported. There was no family history of neurological disease. On examination, a mild expressive dysphasia with a degree of orobuccal apraxia was observed; his speech was prone to error with longer words and he had difficulty in repeating polysyllabic words and multipart sentences, but there was no difficulty with comprehension. There were no visuospatial errors, but he had difficulty with writing and some spelling limitations. There were no parkinsonian signs, but there was slightly jerky pursuit both horizontally and vertically as well as possibly minor dysmetria of saccades. There were occasional limb muscle fasciculations without associated weakness. His Mini‐Mental State Examination was 26/30 and cognitive examination (ACE‐R) score of 84/100. magnetic resonance imaging showed generalized involutional change and several non‐specific high signal lesions. The clinical features favored primary progressive non‐fluent aphasia (PPA) and hexylmethylpropylene amineoxine – single‐photon emission computed tomography scan showed an inferior frontal hypoperfusion, in keeping with PPA in September 2014 (Fig. 1). Nerve conduction studies identified benign fasciculations, but nothing consistent with motor neuron disease (MND). Genetic testing for *C9orf72* was negative. His neurodegenerative condition progressed following presentation, and he developed swallowing difficulties, subsequent aspiration pneumonias as well as seizures. He died at age 71, 10 years after the initial presentation.

## PATHOLOGICAL FINDINGS

According to our brain bank protocol the right half of the brain was sampled fresh and stored in a freezer at −80°C, while the left half of the brain was fixed in formalin and processed for neuropathological examination. The fresh brain weight was 1004 g; the formalin fixed left brain weighed 509 g and the left brainstem and cerebellum hemisphere weighed 90 g. There was mild to moderate generalized cortical atrophy, slightly more prominent in the frontal lobe and around the Sylvian fissure. Mild ventricular dilatation and a slightly smaller hippocampus were also seen.

**Figure 2:**
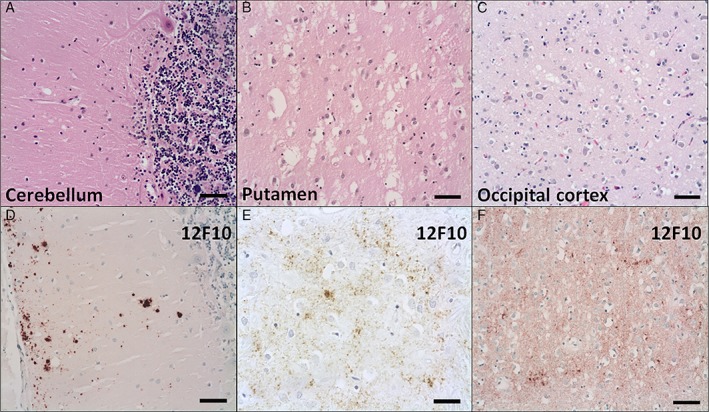
Microphotographs of sections stained with hematoxylin and eosin from the cerebellum (A), putamen (B), and occipital cortex (C) as well as sections immunostained for PrP (12F10) from the cerebellum (D) and parietal (E) and occipital (F) cortices. Some neuronal loss and variable degree of spongiform changes are seen, ranging from a few and isolated microvacuoles in the cerebellum (A) to large coalescent vacuoles in the putamen (B). Transcortical microvacuolation is seen in the occipital cortex (C). PrP immunoreactivity is localized in microplaques in the molecular layer of the cerebellum (D). There is a granular/synaptic pattern in the parietal (E) and occipital (F) cortices, often in a patchy distribution. Scale bars: 50 μm (A‐F).

Histology of the brain revealed mild to moderate neuronal loss in the neocortex, particularly in the frontal and parieto‐occipital lobes. This was associated with microvacuolation, often involving the deeper cortical layers, and no areas of confluent spongiform change were identified (Fig. 2A–C). The microvacuolation was also noticeable in the basal ganglia, medial thalamic nuclei and the cerebellar cortex, while the hippocampus and the brainstem were relatively spared. Despite the neuronal loss, the overall Alzheimer‐type changes were relatively mild by extensive immunohistochemistry. Amyloid‐β (Millipore, Watford, UK) deposition was more widespread, in keeping with Thal phase 3 and Consortium to Establish a Registry for Alzheimer Disease plaque stage A. There was no significant amyloid angiopathy, although a few leptomeningeal blood vessels were labelled by amyloid‐β. The hyperphosphorylated tau (ThermoFisher Scientific, Lutterworth, UK) positive pathology was relatively mild, in keeping with BrainNet Europe (BNE) stage II (NIA‐ABC: A2, B1, C0). Phosphorylated trans‐activation response DNA‐binding protein 43 kDa (ProteinTech, Manchester, UK) and α‐synuclein (BD Biosciences, Wokingham, UK) immunostains were negative.

Immunohistochemistry for PrP showed very similar pattern using the 12F10 (kindly supplied by Professor Hunsmann of the German Primate Centre, Gottingen, Germany) and 3F4 (Dako, Ely, UK) anti‐PrP antibodies, revealing granular/synaptic pattern of accumulation in the cerebral cortex, often in a patchy distribution and particularly in the deeper cortical layers (Fig. 2D–F). There was also labelling in the hippocampus, the basal ganglia and the thalamus. Large numbers of microplaques were seen in the putamen and the cerebellar cortex, particularly within the molecular layer extending up to the subpial regions. No kuru‐type plaques were identified in the cerebellum on hematoxylin and eosin staining or PrP immunohistochemistry.

**Figure 3:**
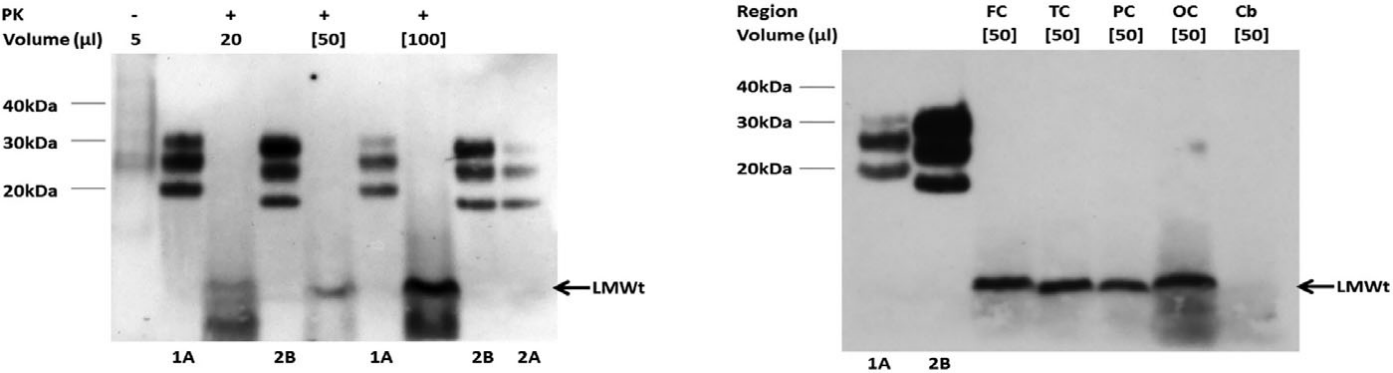
Western blots of PrP in the parietal cortex (left panel) as well as frontal (FC), temporal (TC), parietal (PC), occipital (OC) cortices and cerebellum (Cb) (right panel). Samples from this case is analyzed without (−) and with (+) proteinase K (PK) digestion (50 μg/μL, 1 h, 37°C) (left panel). Samples from different regions are analyzed following PK digestion (right panel). The sample volumes (μL) loaded are indicated; square brackets indicate samples that have undergone centrifugal concentration (each panel). The samples from sCJD MM1 (1A), vCJD (2B) and sCJD VV2 (2A) are compared with PK‐digested standards (each panel). The low‐molecular weight (LMWt) bands evident in this case are marked with arrows.

Analysis of the frozen brain tissue by Western blotting revealed a low molecular weight band in the frontal, temporal, parietal and occipital cortices (Fig. 3). A similar band was present at lesser intensity in the cerebellar cortex and a faint ladder‐like pattern, in the absence of types 1 and 2 isoforms, was also observed in the temporal parietal and occipital cortices. This pattern is diagnostic of VPSPr. Genetic analysis of the codon 129 polymorphism showed valine homozygosity and no mutations in the PrP gene.

## DISCUSSION

Gambetti et al.^5^ in 2008 (USA) reported 11 subjects affected by a distinct prionopathy that differed biochemically and clinically from the other sporadic prionopathies and was characterized by the accumulation of a protease‐sensitive misfolded PrP. Since then, some other cases have been described worldwide, demonstrating a higher incidence than initially thought; further cases of VPSPr have been identified prospectively and retrospectively in the USA,^6^, ^7^ UK,^8^, ^9^, ^10^ the Netherlands,^11^ Austria^12^ and Spain.^13^, ^14^, ^15^ To date altogether 39 VPSPr cases have been published.

Most sporadic prion diseases are characterized by two main PrP^res^ strains that vary from 21 to 19 kDa fragments obtained after PrP^Sc^ proteinase K treatment: types 1 and 2 PrP^res^, respectively. These two subtypes have been experimentally reported to be associated with a variable quantity of another disease‐associated PrP isoform that differs from PrP^c^ and PrP^res^ in that this PrP strain is insoluble but highly sensitive to proteinase K treatment, henceforth referred to as PrP^sen^. Although both sCJD and sporadic fatal insomnia (sFI) reveal types 1 or 2 PrP^res^ isoforms and a small amount of PrP^sen^ on Western blotting, VPSPr is characterized by the presence of various isoforms of PrP^sen^ in the absence of type 1 or 2 PrP^res^ isoforms.^16^ The most interesting aspect of this new disease from a biochemical point of view is that although PrP^Sc^ is abundantly present in the brain, conventional PrP^res^ isoforms are difficult to detect on Western blotting because of their relatively increased sensitivity to proteolysis. PrP^sen^ consists of a C‐ and N‐terminally truncated approximately 8 kDa band that is usually accompanied by a ladder of bands that extend between 18 and 30 kDa in range on Western blotting. Moreover, the ladder‐like pattern appears to be dependent on the codon 129 genotype, which adds more complexity to the biochemical features of VPSPr. Indeed, it has been reported that the electrophoretic migration features of the protease‐resistant bands, and thus the ladder‐like profile intensity on Western blotting, vary depending upon the *PRNP* codon 129 genotypes. It appears that 129MM shows the most intense ladder‐like composition, reflecting a higher PK resistance, whereas 129VV reveals a strong approximately 8 kDa band but faint accompanying ladder‐like bands, hence a lower protease resistance.^6^ The current case revealed biochemical features similar to those found in VPSPr 129VV; *PRNP* codon valine homozygosity in our patient was later confirmed by genetic analysis. Not only is the *PRNP* codon 129 genotype related to a specific electrophoretic pattern, but this genotype also influences the clinical presentation and neuropathological features of VPSPr.^16^

With regard to the clinical presentation, VPSPr appears to be also different from the other sporadic prionopathies. Sporadic prion diseases, such as sCJD and sFI, are clinically characterized by a rapidly progressive dementia and myoclonus^2^ or in the case of sFI, by a progressive insomnia, psychiatric disturbances and a relatively short history of dementia. However, VPSPr shows a much lengthier course of disease than either sCJD or sFI. The presenting symptoms consist of a predominantly fronto‐temporal type of dementia with or without Parkinsonism in the absence of sleep disturbances or involuntary muscle contractions.^16^ The disease progresses toward the development of motor abnormalities, worsening of the initial symptoms and finally enters into an akinetic‐mutism period. Again, depending upon the *PRNP* codon 129 status, the presenting symptoms will vary; 129VV cases (25/37 confirmed VPSPr), usually reveal a more psychiatric, speech and cognitive‐associated initial decline, whereas the 129MM (5/37 confirmed VPSPr) and 129MV (9/38 confirmed VPSPr) counterparts may not be symptomatic at all (two of the MM cases) or present with more parkinsonian features and fewer behavioral disturbances.^6^, ^17^ With regard to the patient described here, the presentation was a long history of language difficulties, with very mild mood disturbances and no signs of parkinsonism or myoclonus. Although there was a minimal memory decline in the beginning, this has only been found to be present at onset in 50% of 129VV cases.^6^ Unlike 129MM and 129MV genotypes, 129VV does not usually present with parkinsonian features, as in our case. However, as the disease progressed, the patient did show gait disturbances which led him to suffer from several falls and also develop other symptoms that have yet not been linked to any of the genotypes, such as seizures or dysphagia. As seen in 50% of 129VV patients, there was no family history of dementia. The patient died approximately 10 years after the onset of the symptoms.

From a neuropathological perspective, the morphology of VPSPr appears to be consistently similar in the three *PRNP* codon 129 genotypes,^6^ but with differences in the intensity of the features. This novel prionopathy is characterized by medium‐sized microvacuolation affecting most of the cerebral areas, variable neuronal loss and distinctive cerebellar molecular layer microplaques. The immunohistochemical PrP pattern also depends on the genotype; it appears to be more severe in 129VV homozygous patients. Our case revealed neocortical neuronal loss, deep and superficial gray matter microvacuolation and cerebellar microplaques. The immunoprofile was consistent with previously published cases of 129VV VPSPr and revealed target and dot‐like immunoexpression with 12F10 and 3F4. In addition, BNE stage II Alzheimer type changes were seen, but no Lewy body pathology was present.

This unexpected case of VPSPr was identified through a brain bank donation and was sampled fresh due to the lack of clinical suggestion of a prionopathy. Several other neurodegenerative conditions were considered prior to the prion immunohistochemistry analysis that allowed the final diagnosis. It is well known that prionopathies, VPSPr included, are transmissible neurodegenerative diseases that pose potential health and safety risks. Although extra health and safety measurements may be advised in suspected prionopathies, it is believed that the standard but rigorous health and safety measures in neuropathology laboratories and brain banks are sufficient to prevent accidental disease transmission. Since VPSPr does not fully follow the typical clinical course for sCJD, it can be expected to occur rarely in future brain bank donations and may cause diagnostic difficulty, particularly if multiple neurodegenerative processes are present. The presence of patchy cerebral cortical spongiform change in the absence of other neurodegenerative pathology should raise the suspicion of VPSPr, even in elderly patients with a lengthy clinical history.

## DISCLOSURE

The authors declare no conflict of interest.
